# Immune-Related Genes to Construct a Novel Prognostic Model of Breast Cancer: A Chemosensitivity-Based Study

**DOI:** 10.3389/fimmu.2021.734745

**Published:** 2021-10-26

**Authors:** Zhi-Min Deng, Wei Hu, Fang-Fang Dai, Meng-Qin Yuan, Min Hu, Yan-Xiang Cheng

**Affiliations:** ^1^ Department of Obstetrics and Gynecology, Renmin Hospital of Wuhan University, Wuhan, China; ^2^ Department of Obstetrics and Gynecology Ultrasound, Renmin Hospital of Wuhan University, Wuhan, China

**Keywords:** chemoresistance, CIBERSORT, TMB, LASSO, WGCNA

## Abstract

Chemotherapy combined with surgery is effective for patients with breast cancer (BC). However, chemoresistance restricts the effectiveness of BC treatment. Immune microenvironmental changes are of pivotal importance for chemotherapy responses. Thus, we sought to construct and validate an immune prognostic model based on chemosensitivity status in BC. Here, immune-related and chemosensitivity-related genes were obtained from GSE25055. Then, univariate analysis was employed to identify prognostic-related gene pairs from the intersection of the two parts of the genes, and modified least absolute shrinkage and selection operator (LASSO) analysis was performed to build a prognostic model. Furthermore, we investigated the efficiency of this model from various perspectives, and further validation was performed using the Cancer Genome Atlas (TCGA) cohorts. We identified seven immune and chemosensitivity-related gene pairs and incorporated them into the Cox regression model. After multilevel validation, the risk model was found to be closely related to the survival rate, various clinical characteristics, tumor mutation burden (TMB) score, immune checkpoints, and response to chemotherapeutic drugs. In addition, the model was verified to exhibit predictive capacity as an independent factor over other candidate clinical features. Notably, the constructed nomogram was more accurate than any single factor. Altogether, the risk score model and the nomogram have potential predictive value and may have important practical implications.

## 1 Introduction

Breast cancer (BC) is the most prevalent cancer type and it is the principal cause of cancer-related death in women. BC is highly curable when diagnosed early and treated appropriately (1). Currently, comprehensive modality therapy, combining local treatment (surgery and radiotherapy) with systemic therapy (endocrine therapy, chemotherapy, etc.), is a relatively well-established treatment for BC (2). Among these, chemotherapy is an important treatment modality. Nevertheless, chemotherapy resistance always leads to the failure of chemotherapy and the relapse of cancer, and it remains a significant barrier for the treatment of BC patients. Therefore, additional studies are urgently needed to overcome this obstacle and develop anti-resistance strategies.

Much effort has been made to uncover the mechanisms of chemoresistance and identify novel molecular targets in recent years. Of these, immune checkpoint inhibitors (ICIs) have attracted tremendous attention in overcoming drug resistance because of their properties that harness patient’s own immune system to selectively target and kill cancer cells (3). Recent studies revealed that ICIs were involved in the progression of BC (4), and blocking immune checkpoints can even increase the sensitivity to chemotherapeutic drugs (5). Not only that, studies have proven that tumor-associated macrophages (TAMs) and CD8^+^ T lymphocytes within tumor immune microenvironment are also critically associated with chemoresistance (6, 7). Altogether, existing evidence suggests that the tumor immune microenvironment inevitably interacts with the mechanisms of chemoresistance while limiting the antitumor immune response (3, 8, 9). Thus, predicting the evaluation of treatment response and prognosis in BC patients and taking the immune infiltration signature of tumors into consideration at the same time would be of great clinical interest.

Currently, various kinds of models focusing on the immune infiltration signature of tumors showed superior predictive performance in BC (10–12). However, few models have previously combined chemosensitivity and immune signatures. We believe that evaluating chemosensitivity and tumor immune infiltration at the same time may have a better predictive value. Moreover, models constructed with two-biomarker combinations, such as gene pairs, may have higher accuracy than those constructed with a single marker, and no specific expression data should be required. Given this, the aim of this study was to build a prognostic model for BC constructed from immune-related and chemosensitivity-related gene pairs. Then, the evaluation was performed from various dimensions, including survival rate, immune checkpoints, and the response to chemo drugs.

## 2 Materials and Methods

### 2.1 Identification of Differentially Expressed Genes Associated With Chemosensitivity Status

The normalized gene expression profiles of GSE25055, an expression profile chip of BC from GEO (http://www.ncbi.nlm.nih.gov/geo/), were prepared using the *GEOquery* package in R 4.0.2. This chip, performed on the GPL96 platform, comprises 113 samples with chemosensitivity and 197 insensitive samples. Then, standard steps, including converting the probe names into gene names, log 2 transformation, and removing duplicate probes, were performed for initial processing.

Gene set enrichment analysis (GSEA) was utilized to obtain the cognitive pathways most affected by chemosensitivity-related genes in the GSE25055 datasets with the *clusterProfilter* package. We set the cutoff criterion to adj. *p* < 0.05. Next, visualization of the interesting results, predominantly immune pathways and cancer-related pathways in two parts, was performed using the *enrichplot* package. Subsequently, the *limma* package was applied to screen out the differentially expressed (DE) genes between the chemosensitive and insensitive groups in the GSE25055 dataset to obtain the DE chemosensitivity-related genes (DECRGs). *p* < 0.05 and log 2-fold fold change (logFC) > 0.5 served as the cutoff criteria.

### 2.2 Screening Differentially Expressed Chemosensitivity-Related and Immune-Related Genes and Performing Enrichment Analysis

To obtain the immune phenotype-relevant genes, first, single sample gene set enrichment analysis (ssGSEA) and GenePattern environment-based analysis were employed to evaluate the immune infiltration level (recorded as the ssGSEA score) in each sample (13). Next, unsupervised hierarchical clustering analysis of the ssGSEA output matrix was carried out using the *ConsensusClusterPlus* package to obtain the optimal immune grouping (the *K* value), and the range of *K* values was set from 2 to 5 (14). Furthermore, the DE immune-relevant genes (DEIRGs) were calculated across subgroups by the *limma* package with the same cutoff criteria as described for the process of screening out DECRGs. In general, investigation of immune-related genes *via* the method described above instead of downloading directly from the ImmPort database, a human immunological database, results in higher accuracy and precision (15).

Through the above analytic steps, the DECRGs and DEIRGs were collected. Afterward, the intersection of the genes from the two parts was crossed to obtain the DECRGs that were immune-relevant at the same time (DECIRGs), and the online tool Venny 2.1 (https://bioinfogp.cnb.csic.es/tools/venny/index.html) was utilized for visualization. Gene Ontology (GO) and Kyoto Encyclopedia of Genes and Genomes (KEGG) enrichment analyses were carried out using the R package *clusterProfiler*. The inclusion criteria were as follows: *p*-value < 0.05 and *q-*value < 0.05.

### 2.3 Validation of the Plausibility of the Immune Phenotype Grouping

The tumor microenvironment (TME) of GSE25055, including immune and stromal scores, was calculated using the ESTIMATE algorithm. Visualization was implemented by the *pheatmap* and *ggpubr* packages.

Subsequently, cell-type identification by estimating the relative subset of known RNA transcripts (the CIBERSORT algorithm), a common method used for the evaluation of immune cell infiltration, was performed to quantify the immune cell proportion with 1,000 permutations. Only samples with a *p*-value < 0.05 were included.

In addition, human leucocyte antigen (HLA), a set of linkage gene groups, is widely applied in the field of immune-related diseases (16). Consequently, the expression matrix of *HLA* genes was extracted from GSE25055.

The above three methods, tumor microenvironment score, immune cell infiltration score, and *HLA* gene expression matrix, were chosen to validate the plausibility of immune grouping. The latter two were graphed as boxplots by the *ggpubr* package.

### 2.4 Construction of the Risk Assessment Model Based on the DECIRG Pairs

In the majority of previous studies, the construction of a risk assessment model or immune score prognostic model was based on an expression matrix of screened genes. The clinical application of such a strategy is significantly hindered by the various sources of the expression matrix of genes, including gene chip data and quantitative reverse transcription-PCR (qRT-PCR) data. These data need batch correction before applying the model, which undoubtedly increases the workload.

Thus, here, a 0-or-1 matrix was constructed through an iterative loop to cyclically pair the DECIRGs. The representation of the paired sample ID was “A|B”. When the expression level of gene A was higher than that of gene B, the pair was presented as “1”; otherwise, it outputted “0”. With this strategy, only the relative expression of the genes was under consideration without dwelling on the absolute figures. Additionally, only samples with a 0.2–0.8 pair ratio, defined as the total pair value/sample numbers ratio, were included. Furthermore, after combining the survival data taken from the GEO database, univariate analysis was computed using Cox regression.

Next, least absolute shrinkage and selection operator (LASSO) regression was run 1,000 times with the *glmnet* package to preliminarily screen the DECIRG pairs, and then Cox proportional hazard regression was used to further reduce the number of pairs to build a risk assessment model. *p* < 0.05 was set as the inclusion criteria. A formula for the risk model was established for all patients:


$$\text{Riskscore}=\Sigma_{i=1}^{k}\;\text{Cox coefficient of pair }i\times \text{Expression value of pair }i$$

Moreover, the maximum area under the curve (AUC) was calculated, and the inflection point with the largest sum of sensitivity and specificity was identified as the optimal cutoff point (opcut).

### 2.5 Evaluation of the Risk Model

Before evaluation of the risk model, the clinical data from GSE25055 and the somatic mutation and clinical and gene expression data of BRCA (breast invasive carcinoma) in The Cancer Genome Atlas (TCGA) were downloaded and initially processed. Of note, those samples whose follow-up was 0 days were culled. The same cyclical pair treatment was performed for the DECIRGs in TCGA to obtain the DECIRG pairs as the validation set.

To determine the performance of the constructed model, different aspects of the model have been assessed. First, survival curves were drawn by the *survival* package using the log-rank test and Kaplan–Meier (K-M) method to compare the survival difference between the high-risk and low-risk groups. In addition, the grouping condition of patients and the survival state per case were plotted. Second, the independent prognostic value of the factors was computed by univariate and multivariate analysis. If the *p*-value of one factor < 0.05, it means this factor can be used as an independent predictor of survival. After that, receiver operating characteristic curves (ROCs) were used to evaluate the accuracy of the risk model (including the sensitivity and specificity).

### 2.6 Exploration of the Value of the Clinical Evaluation by the Risk Model

To further enhance the value of practical applications of the risk score model, independent risk factors identified by previous multivariate analysis was applied to construct a nomogram that combined the risk score and the clinical features. Moreover, the concordance index (C-index) was utilized to measure the accuracy of the nomogram, and calibration curves were plotted to assess the calibration of the models. Then, the ROC curves of various clinical characteristics were drawn, and the AUC was calculated. Subsequently, a series of chi-square tests were applied to uncover the relationship between the risk score and the clinicopathological features by the Wilcoxon signed-rank test.

In general, ROC curves and AUC values are used to judge the performance of a prognostic model. Nevertheless, this strategy pursues accuracy, which does not always equate to the maximum benefit for patients. Herein, decision curve analysis (DCA) was employed to estimate the clinical benefits by logistic regression analysis. More than those, Cox regression was also applied to draw the DCA curves for taking the prognosis of patients under consideration. In these operations, the R packages *survival*, *rms*, *survivalROC*, *rmda*, and *stdca* were utilized.

### 2.7 The Correlation Between the Risk Subgroups and the Chemo Drugs, Immune Checkpoints, and Somatic Mutations

The linkages between the risk grouping and some common chemotherapy agents for BC, for example, cisplatin, vinblastine, docetaxel, cyclopamine, doxorubicin, and gemcitabine, were primarily investigated since our study focused on chemosensitivity-based biomarkers. In this procedure, the *limma*, *pRRophetic*, and *ggplot2* packages were used.

In addition, immune-related phenotypes were also the theoretical basis of our research. Immunotherapy, represented by immune checkpoints (ICs), has made great progress in the entire tumor area in recent years, and BC is no exception. Therefore, the expression levels of ICs, including *PDCD1* (PD-1, programmed cell death protein 1), *LAG3* (lymphocyte activation gene 3 protein), *CTLA4* (cytotoxic T-lymphocyte-associated protein 4), *IDO1* (indoleamine 2,3-dioxygenase), and *CD27* (Cluster of Differentiation 27), between the high-risk and low-risk groupings were measured.

In the third part, somatic mutation data of patients in the TCGA cohort were obtained to investigate the mutation differences among the risk groupings. Then, the *maftools* package was applied to perform the analysis of the tumor mutation burden (TMB) of TCGA, calculate the TMB score, and draw the waterfall plot. Whether the TMB score is related to the risk score and patient survival probability was then explored. Based on the median value of the TMB score as a cutoff, the samples were divided into two groups, high-TMB and low-TMB, and integrated with their corresponding risk groupings. The correlation between the risk cluster and the TMB cluster was determined by Pearson correlation analysis.

### 2.8 Screening and Validation of Hub DECIRGs Pairs

To improve understanding of association between the risk score model and molecular clusters on the one hand, and screening the hub gene pairs on the other hand, Weighted Gene Co-Expression Network Analysis (WGCNA) was utilized. In detail, based on the risk score, we first conducted the sample clustering and calculated the power value. Then, the correlation coefficient and the best soft power were filtered out using the *WGCNA* package in R 4.0.2 (17). Modules with high correlation coefficient were selected for further analysis. Furthermore, the hub gene pairs were obtained by taking the intersection of selected WGCNA modules above and the seven pairs of DECIRGs.

To further determine the expression level of the hub gene pairs, immunohistochemical (IHC) images of all single genes were downloaded from the Human Protein Atlas (HPA) database (https://www.proteinatlas.org/). Subsequently, clinical cancer samples and adjacent paracancer tissues from three patients with BC were collected at the Renmin Hospital of Wuhan University (Wuhan, China). Total RNA was isolated with Trizol from tissues using the Nucleospin RNA II Kit (Servicebio). Then, the reversed transcription was carried out by qRT-PCR kit (Servicebio). In this progress, relative gene expression was standardized to GAPDH. The primers for target gene pairs and internal reference gene are listed in **Supplementary Table S1**. In addition, data were processed using the comparative Ct (2^−ΔΔCT^) method and each sample was repeated at least three times.

## 3 Results

### 3.1 Relationship of Chemosensitivity and Immune Status in BC Patients

**Figure 1:**
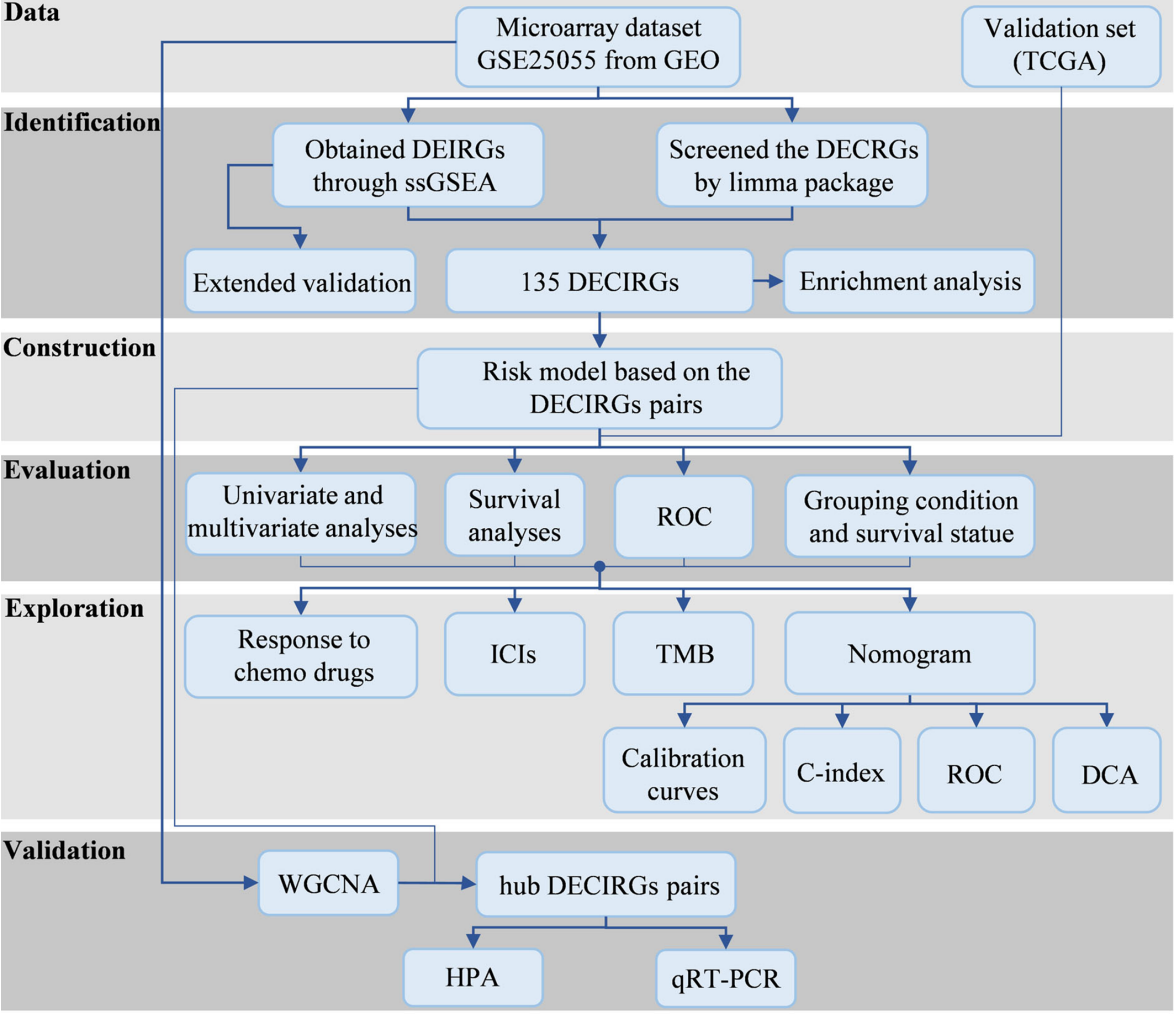
The study processes. GEO, Gene Expression Omnibus; TCGA, The Cancer Genome Atlas; DEIRGs, differentially expressed immune-relevant genes; DECRGs, differentially expressed chemosensitivity-related genes; ssGSEA, single sample gene set enrichment analysis; DECIRGs, differentially expressed chemosensitivity-related and immune-related genes; ROC, receiver operating characteristic curves; ICs, immune checkpoints; TMB, tumor mutation burden; C-index, concordance index; DCA, decision curve analysis; WGCNA, Weighted Gene Co-Expression Network Analysis; HPA, Human Protein Atlas; qRT-PCR, quantitative reverse transcription-PCR.

**Figure 2:**
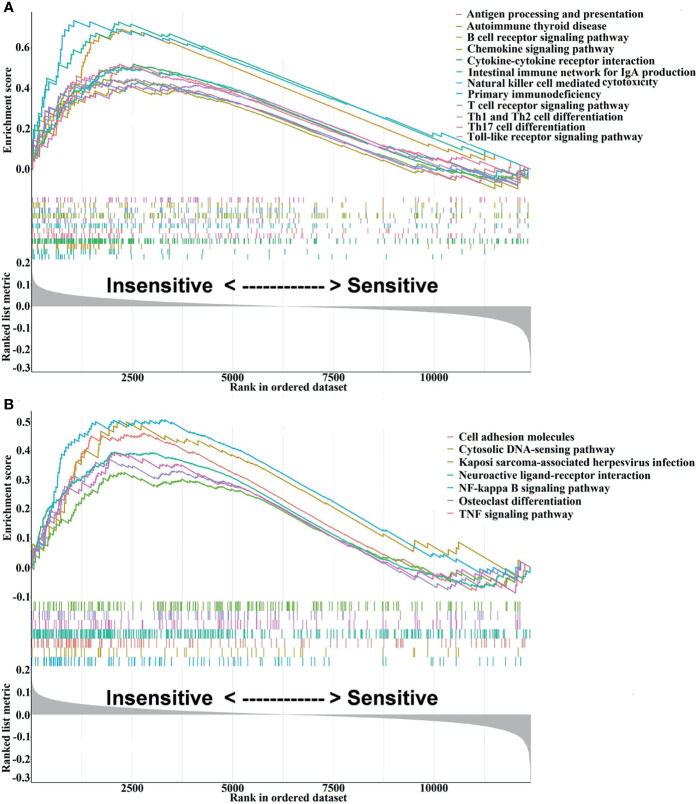
Visualization of the interesting results, including 12 immune-related and 7 tumor-related signaling pathways, was performed using the *enrichplot* package. **(A)** The top 12 immune-related signaling pathways. **(B)** The top 7 tumor-related signaling pathways.

Flow chart of the study design is depicted in **Figure 1**. The GSEA results are presented in **Supplementary Table S2**, from which 48 signaling pathways were enriched between the chemosensitive and insensitive subgroups based on the cutoff criteria of adj. *p* < 0.05. **Figure 2** shows that the top 12 immune-related signaling pathways (**Figure 2A**), including antigen processing and presentation, B-cell receptor signaling pathway, and chemokine signaling pathway, and the top 7 tumor-related signaling pathways (**Figure 2B**), such as cell adhesion molecules, neuroactive ligand–receptor interaction, and TNF signaling pathway, were visualized according to their enrichment score. The findings listed above indicate that the chemo-insensitive status in BC patients is not only related to the proliferation, invasion, and metastasis of tumors but also related to the activity of the immune response and tumor immune infiltration. Herein, we can reasonably conclude that the relationship between chemosensitivity and immune status in BC patients is intense.

Additionally, differential gene expression analysis comparing chemosensitive *versus* insensitive samples yielded 262 significantly DECRGs (*p* < 0.05, logFC > 0.5).

### 3.2 The Grouping of GSE25055 on the Basis of the Immune Landscape and Then Obtaining the DECIRGs

Due to space limitations, the immune infiltration level of a portion of the samples, recorded through the ssGSEA method, is displayed in **Supplementary Table S3** (five samples, 29 immune cell types).

**Figure 3:**
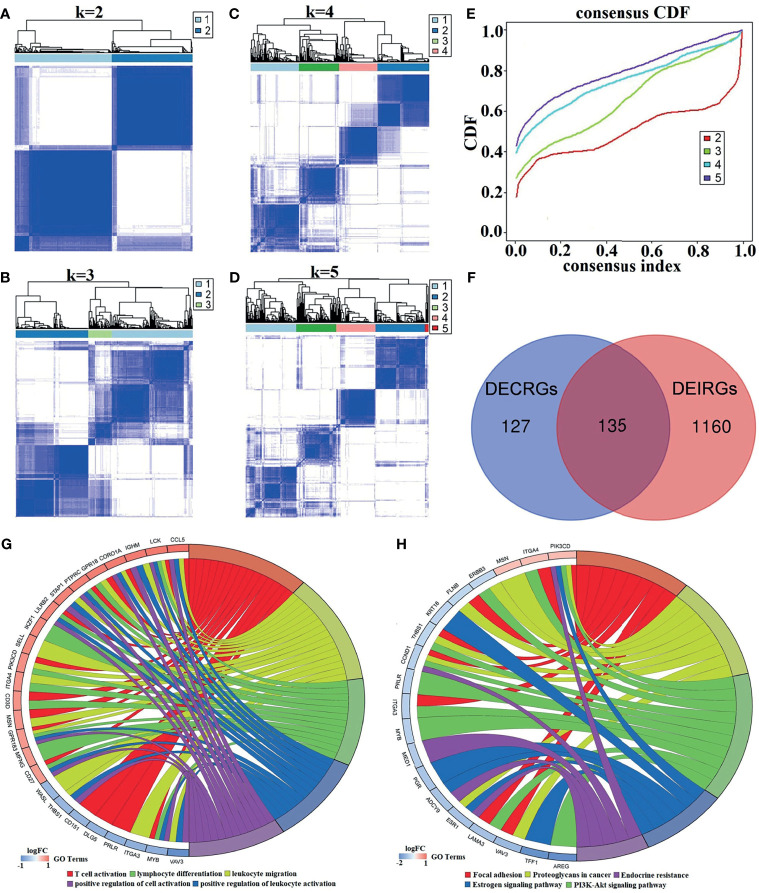
The process of identifying *K* values and DECIRGs and the results of the enrichment analysis. **(A–D)** The consensus matrix (CM) plots. **(A)** *K* = 2; **(B)** *K* = 3; **(C)** *K* = 4; **(D)** *K* = 5. **(E)** The cumulative distribution function (CDF) plots. **(F)** Venn plot of the intersection of DECRGs and DEIRGs. **(G)** The GO enrichment results of the 135 DECIRGs (only the top 5 items). **(H)** The KEGG enrichment results of the 135 DECIRGs.

Generally, unsupervised hierarchical clustering analysis can extract the features of samples and further classify them in the case of samples without labels. In our study, the immune infiltration level for each sample was obtained, and the groupings of the samples had unclear opposite results. For this reason, combined consensus clustering and PAC were used to test the optimal value of *K*. As clarified in the consensus matrix (CM) plots (**Figures 3A–D**), the clustering effect varies by the *K* value, and the squares share the darkest blue and the least noise when *K* is 2 (**Figure 3A**). Cumulative distribution function (CDF) plots, which display the cumulative consensus distributions for each *K*, indicate that the slope of the decline of CDF was considerably weaker when *K* equals 2 (**Figure 3E**). It can be seen from the tracking plot (**Supplementary Figure S1A**) that the ordinate represented the grouping situation (*K* = 2–5), while the abscissa indicates the different samples. The colors in **Supplementary Figure S1A** represent different subclusters, and the samples with the same color have the same immune properties. The *y*-axis of the delta area plot (**Supplementary Figure S1B**) and the relative change of the area under the CDF curve indicates the relative increase in cluster stability and further supports the finding that the inflection point 2 is the most valuable.

All of the above results revealed that the optimal number of immune classifications was 2, which means that two distinct patterns of BC samples from GSE25055 can be observed: one with low immunity (Immunity-L) and the other with high immunity (Immunity-H). After unsupervised hierarchical clustering analysis, at a cutoff of *p* < 0.05 and logFC > 0.5, 1,295 significant DEIRGs were identified from the two groups. Moreover, in **Figure 3F**, 135 DECIRGs are obtained from taking the intersection of 262 DECRGs and 1295 DEIRGs. The detailed DECIRGs are provided in **Supplementary Table S4**. Moreover, GO enrichment analysis of the 135 DECIRGs revealed that they were involved in immune-relevant biological processes such as T-cell activation, leukocyte migration, and cell adhesion (**Figure 3G** and **Supplementary Table S5**). The top 5 pathways are displayed in **Figure 3H** and **Supplementary Table S6**, indicating that they participate in proteoglycans in cancer, the estrogen signaling pathway, endocrine resistance, and the PI3K-Akt signaling pathway.

### 3.3 Validation of the Feasibility of the Immune Grouping Strategy

**Figure 4:**
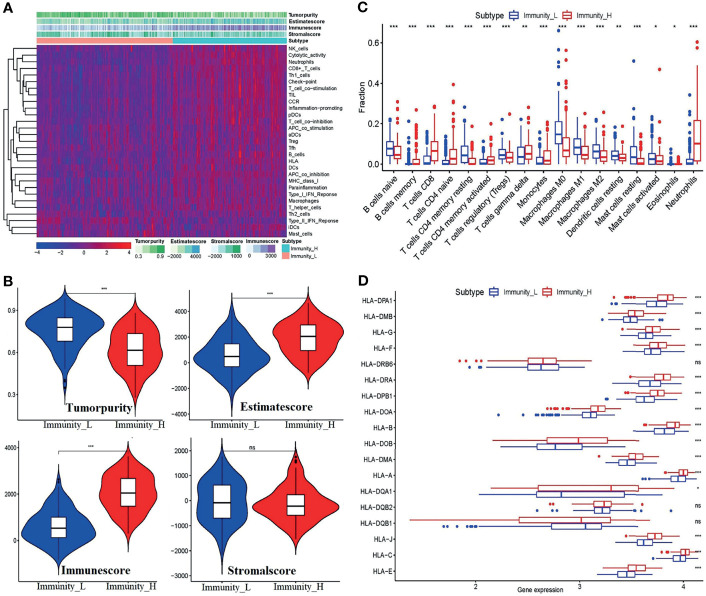
Validation of the feasibility of the immune grouping strategy. **(A)** Heatmap of tumor purity, ESTIMATE score, immune score, and stromal score. **(B)** Violin plots showing the comparisons of the tumor purity, ESTIMATE score, immune score, and stromal score between the Immunity-L and Immunity-H subtypes. **(C)** The fraction of 17 kinds of immune cell infiltration scores between the two groupings. **(D)** Boxplot of *HLA* gene expression between the two groups. *p < 0.05, **p < 0.01,***p < 0.001, ns, no significant differences.

To further uncover the above immune heterogeneity between groupings, ESTIMATE algorithms were performed with R packages. **Figures 4A, B** show the tumor microenvironment score analysis results of BC. From the figure, it can be seen that in the Immunity-H group, the immune score and ESTIMATE score were markedly enhanced, whereas the tumor purity was well below that in the Immunity-L group. Concomitantly, the stromal score is not visibly higher. Similarly, the immune cell infiltration level per sample was measured by the CIBERSORT algorithm. As shown in **Figure 4C**, the infiltration scores of 17 kinds of immune cells contrasted starkly between the two groups. Alternatively, the boxplot of the expression level of the *HLA* genes (**Figure 4D**) shows that except for *HLA-DR* beta 6 (*HLA-DRB6*), *HLA-DQB2*, and *HLA-DQB1*, the expression of the *HLA* genes depicts distinguishable differences between the two groups.

These results indicated that the strategy of combining ssGSEA with unsupervised hierarchical clustering analysis to divide the samples into the Immunity-L group and Immunity-H group is reasonable. Generally, in the Immunity-H group, the activity of immune responses, expression of the majority of *HLA* genes, and the level of the immune score and ESTIMATE score increased, and tumor purity decreased correspondingly.

### 3.4 Construction and Valuation of the Risk Score Model for DECIRG Pairs

Through an iterative loop, a total of 3,268 DECIRG pairs in 309 GEO samples were obtained and further reduced to 1,232 pairs by univariate Cox analysis. One sample was excluded due to missing follow-up data. Likewise, in the TCGA cohort, 3,087 pairs in 692 samples were included.

**Figure 5:**
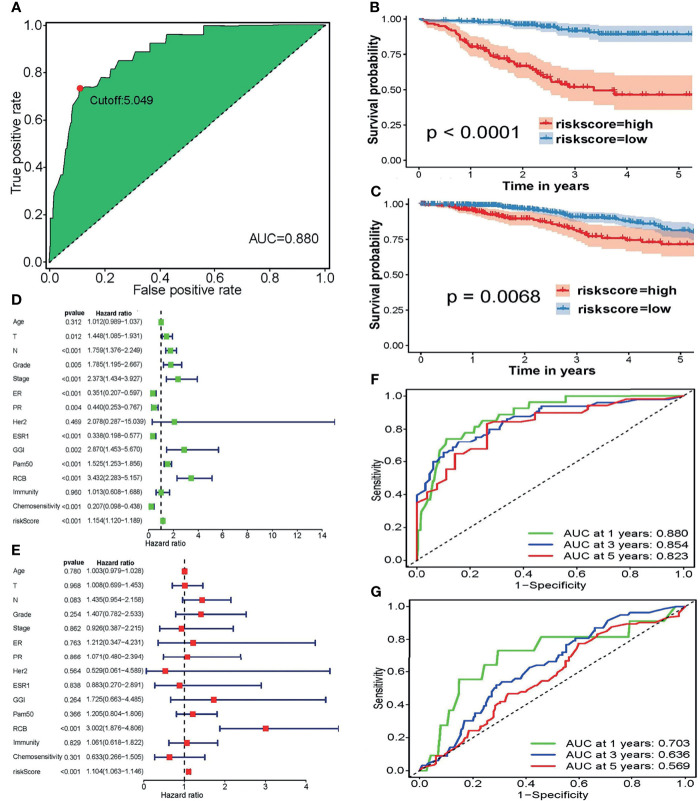
**(A)** The optimal cutoff points in the ROC curve with the maximum AUC. **(B, C)** The survival curves to estimate the difference in the survival time between the high-risk and low-risk groupings in the **(B)** training and **(C)** validation sets. **(D, E)** Forest plots of the training set using **(D)** univariate and **(E)** multivariate independent prognostic analyses. **(F, G)** To evaluate the accuracy of our risk model, 1-, 3-, and 5-year ROC curves were plotted in the **(F)** GEO and **(G)** TCGA datasets.

Depending on the LASSO regression method, 20 pairs of DECIRGs were obtained (**Supplementary Table S7**), and then the pairs were reduced to 8 after the Cox proportional hazard regression, and 7 pairs of DECIRGs finally served as the foundation of the risk score model (*p* < 0.05). See **Table 1** and **Supplementary Figures S2A, B** for details. The computational method of the risk score for each patient is described in the *Materials and Methods* section. **Figure 5A** shows that the opcut is 5.049. Next, 258 low-risk and 51 high-risk samples were obtained in the training set (GEO database). At the same time, in the TCGA validation set, 587 samples were classified into the low-risk group, and the remaining 105 were classified into the high-risk group. The detailed clinical information of the patients included in the training and validation datasets is shown in **Table 2**.

**Table 1:** The seven pairs of DECIRGs that were used to build the risk score model.

Pair ID	Coef	HR	HR.95L	HR.95L	*p*-value
LCK|APBA2	−1.05596	0.347859	0.204894	0.59058	9.23E-05
MSN|CD151	0.841157	2.319049	1.175758	4.574063	0.015217
ITGA4|NAT1	0.606283	1.833603	1.032612	3.255918	0.038498
ST8SIA4|PSD3	0.705933	2.025737	1.211581	3.386986	0.007107
PEX11A|GREB1	0.690695	1.995101	1.183866	3.362229	0.009492
ACACA|RABEP1	0.866912	2.37955	1.33674	4.235871	0.003215
ACOX2|AREG	0.767286	2.153912	1.265167	3.666974	0.004708

Coef, coefficient; HR, hazard ratio.

**Table 2:** Detailed clinical information of the patients included in the training and validation datasets.

Category	GEO (*n* = 279)		TCGA (*n* = 447)
OS (years ± SD)	2.88 ± 1.72		3.58 ± 3.27
Age (years ± SD)	50.38 ± 10.70		57.65 ± 12.87
Death	59 (21.15%)		66 (14.77%)
Alive	220 (78.85%)		381 (85.23%)
TNM-T0/T1	21 (7.53%)		117 (26.17%)
TNM-T2	153 (54.84%)		262 (58.61%)
TNM-T3	61 (21.86%)		47 (10.51%)
TNM-T4	44 (15.77%)		21 (4.70%)
TNM-N0	79 (28.32%)		168 (37.58%)
TNM-N1	133 (47.67%)		181 (40.49%)
TNM-N2	37 (13.26%)		70 (15.66%)
TNM-N3	30 (10.75%)		28 (6.26%)
TNM-M0	–		436 (97.54%)
TNM-M1	–		11 (2.46%)
Grade-1	18 (6.45%)		–
Grade-2	109 (39.07%)		–
Grade-3	142 (50.90%)		–
Grade-4	10 (3.58%)		–
Stage-I	6 (2.15%)		67 (14.99%)
Stage-II	151 (54.12%)		246 (55.03%)
Stage-III	122 (43.73%)		122 (27.29%)
Stage-IV	–		12 (2.68%)
HER2-Positive	3 (1.08%)		66 (14.77%)
HER2-Negative	276 (98.92%)		381 (85.23%)
ESR1-Positive	156 (55.91%)		–
ESR1-Negative	123 (44.09%)		–
PR-Positive	126 (45.16%)		302 (67.56%)
PR-Negative	153 (54.84%)		145 (32.44%)
ER-Positive	158 (56.63%)		350 (78.30%)
ER-Negative	121 (43.37%)		97 (21.70%)
PAM50-Normal	23 (8.24%)		9 (2.01%)
PAM50-LumA	87 (31.18%)		217 (48.55%)
PAM50-LumB	41 (14.70%)		111 (24.83%)
PAM50-Basal	111 (39.78%)		78 (17.45%)
PAM50- Her2	17 (6.09%)		32 (7.16%)
GGI-High	185 (66.31%)		–
GGI-Low	94 (33.69%)		–
RCB-0/I	82 (29.39%)		–
RCB-II	124 (44.44%)		–
RCB-III	73 (26.16%)		–
Immunity-Low	140 (50.18%)		–
Immunity-High	139 (49.82%0		–
Chemosensitivity-Insensitive	178 (63.80%)		–
Chemosensitivity-Sensitive	101 (36.20%)		–
Riskscore-Low	231 (82.80%)		319 (71.36%)
Riskscore-High	48 (17.20%)		128 (28.64%)

**Figures 5B, C** presents the excellent prognostic value of the risk model in both the training and validation sets. Patients with low-risk scores experience a significant enhancement in mean survival time in both the GEO and TCGA datasets compared with those at high risk. The heatmap of the grouping condition and the survival status scatter plot of each case are shown in **Supplementary Figures S2C–F**. We can see that the risk score model can sharply distinguish the surviving and nonsurviving patients in the training set, while their differentiation capacities are weaker in the validation set.

**Figure 5D**, showing forest plots of the training set using univariate analyses, indicates that lymph node (N) TNM stage, tumor grade, FIGO stage, genomic grade index (GGI), residual cancer burden (RCB) score, and risk score are risk factors. In contrast, the positive expression of estrogen receptor (*ER*) and progesterone receptor (*PR*), mutation of the *ESR1* (estrogen receptor-alpha) gene, and a sensitive response to chemotherapy are protective factors. Correspondingly, only the RCB score and risk score was verified as risk factors after multivariate independent prognostic analyses (**Figure 5E**). The details of the univariate or multivariate independent prognostic analyses are displayed in **Supplementary Table S8**. More remarkably, neither separate immunity nor separate chemosensitivity can be treated as independent predictors, whereas the risk score combining immunity with chemosensitivity can be treated as an independent predictor. Therefore, we conclude that regardless of whether univariate or multivariate independent prognostic analyses are performed, the risk score model is an independent prognostic factor.

To evaluate the accuracy of our risk model, the 1-, 3-, and 5-year ROC curves plotted by the *timeROC* package are presented in **Figures 5F, G**, which demonstrates that the accuracy of the model’s prediction is excellent in the GSE25055 dataset. Although the prediction effect was worse in the TCGA cohort, all AUC values > 0.5.

In summary, based on the results described above, the risk score model is considered to be an independent prognostic factor and it has a relatively good accuracy.

### 3.5 The Clinical Evaluation Ability of the Risk Model

**Figure 6:**
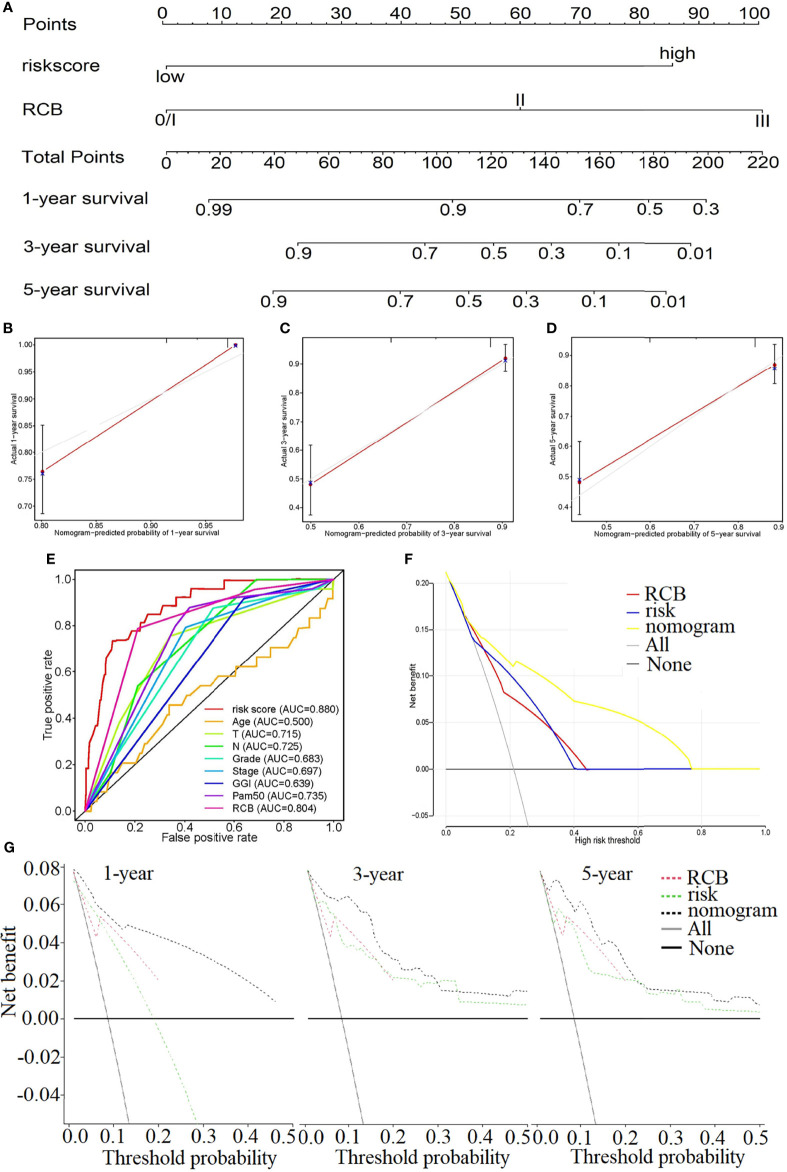
**(A)** Nomogram to predict the 1-, 3-, and 5-year survival rate in GSE25055. **(B–D)** Calibration plots of the nomogram to predict the survival rate at 1 **(B)**, 3 **(C)**, and 5 years **(D)**. **(E)** The comparison of the ROC curve with the other clinical characteristics highlights the superiority of our risk score model. The decision curve plots of the “nomogram”, “RCB”, “risk”, “All” and “None” models by logistic regression **(F)** and Cox regression analysis **(G)**.

Due to the results of the previous step, the RCB and risk score were introduced into the construction of the nomogram, in which the risk score weighs heavily in the total points (**Figure 6A**). The C-index of the nomogram is 0.830. The calibration diagrams, **Figures 6B–D**, state that the predicted survival rate (the red line) at 1, 3, and 5 years was very close to the actual survival rate (the gray line). Additionally, the comparison of the ROC curve with the other clinical characteristics highlights the superiority of our risk score model, whose AUC value is the maximum (**Figure 6E**). The relationship between the risk score and the clinical characteristics is investigated in **Supplementary Figures S3A–L**, which illustrates that the risk score is strongly associated with those characteristics except for the phenotype of *HER2* (**Supplementary Figure S3H**).

**Figures 6F, G** show the decision curve plot. The lines in the figure marked “All” and “None” represent two extreme conditions. When the abscissa, the high-risk threshold, is in the range of approximately 0.0–1.00, the net benefit (ordinate) of the nomogram model is consistently higher than that of the risk and RCB models. That is, the nomogram represents better benefits for patients compared with any single factor. This conclusion was also verified at multiple time points (1, 3, and 5 years).

### 3.6 Exploration of the Potential Relevance of the Risk Score With Chemotherapy Drugs, ICs, and TMB

**Figure 7:**
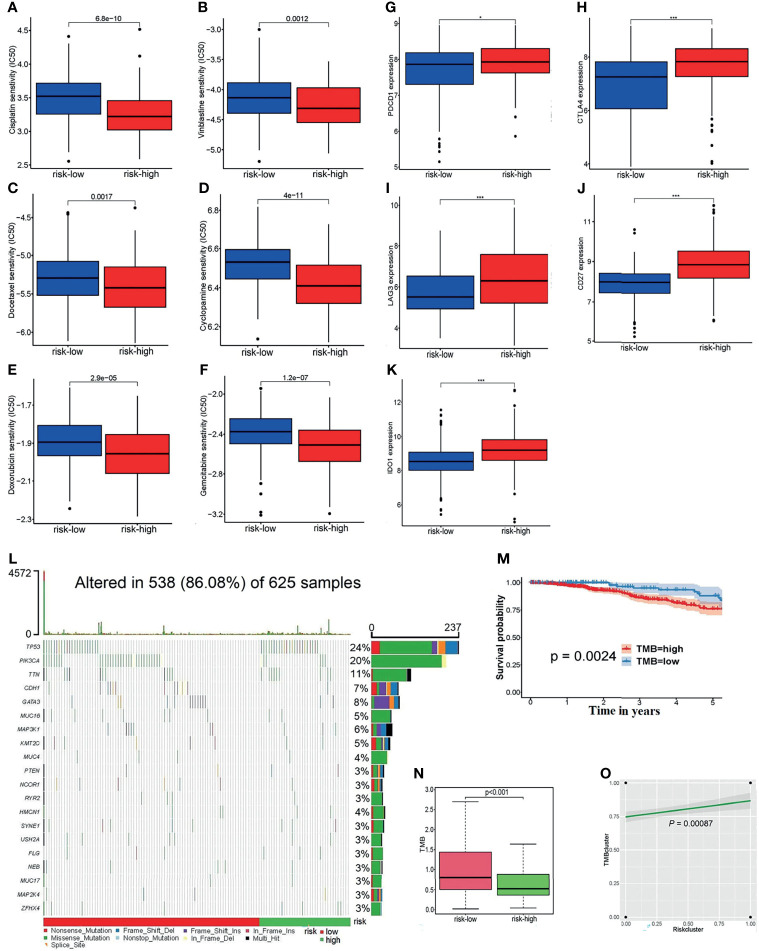
**(A–F**) The linkages between the risk grouping and some common chemotherapy agents for BC, for example, **(A)** cisplatin, **(B)** vinblastine, **(C)** docetaxel, **(D)** cyclopamine, **(E)** doxorubicin, and **(F)** gemcitabine. **(G–K)** Analyses of the relationship of ICs with the risk score, including **(G)** *PDCD1*, **(H)** *CTLA4*, **(I)** *LAG3*, **(J)** *CD27*, and **(K)** *IDO1*. **(L)** The waterfall diagram of the altered genes in 625 samples. **(M)** The survivorship curve indicates that patients with low TMB scores have a longer survival time (*P* = 0.0024). **(N)** The boxplot reflects the correlation between the risk cluster and the TMB score (*p* = 0.0024). **(O)** There was a positive association between the risk clusters and TMB clusters (*p* = 0.00087).*p < 0.05, ***p < 0.001.

The boxplots, **Figures 7A–F**, display that as the grouping of the risk score varies, so does the half-maximal inhibitory concentration (IC_50_) of some chemotherapeutic drugs. From the figure, the IC_50_ of samples with high-risk scores are much lower than that of the low-risk samples. The higher the risk score, the lower the sensitivity to chemotherapeutic drugs such as cisplatin, vinblastine, docetaxel, cyclopamine, doxorubicin, and gemcitabine. This suggests that the risk model may serve as a potential tool to predict the patient’s responsiveness to chemotherapy agents.

In addition, the expression of common ICs is shown in **Figures 7G–K**, which reveals that the risk score is positively correlated with the expression levels of *PDCD1, CTLA4, LAG3, CD27*, and *IDO1* between the high-risk and low-risk groups.

Then, the somatic mutation data of 625 samples were downloaded from the TCGA-BRCA databases. The waterfall diagram expresses the rank of the genes’ mutation frequency in those samples (**Figure 7L**). It can be seen from the legend that the high-risk patients had a high gene mutation frequency. According to the TMB score, 489 high-TMB and 136 low-TMB samples were obtained. The survivorship curve indicated that patients with low TMB scores had a longer survival (*P* = 0.0024) (**Figure 7M**). **Figure 7N** shows that the low-risk patients usually shared lower TMB scores; at the same time, there was a positive association between the risk clusters and TMB clusters (*P* = 0.00087) (**Figure 7O**).

### 3.7 Identification and Exploration of Hub DECIRGs Pairs

**Figure 8:**
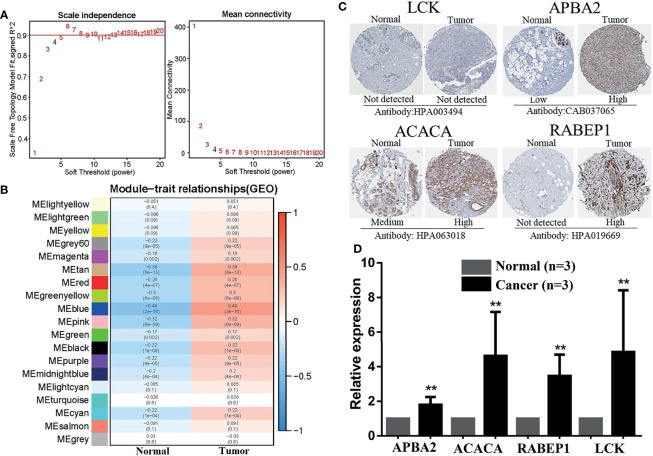
Identification and exploration of hub DECIRGs pairs. **(A)** Determination of soft-thresholding power. When soft threshold comparable to 4, *R* ^2^ > 0.8 and mean connectivity < 100, the network was scale-free topology. **(B)** Module–trait associations: Each row corresponds to a module and each column to a trait (low-risk and high-risk). **(C)** The IHC staining images of *LCK*, *APBA2*, *ACACA*, and *RABEP1*. **(D)** The mRNA expression analysis of *LCK*, *APBA2*, *ACACA*, and *RABEP1* by qRT-PCR. **p < 0.01.

A total of 3,268 gene pairs were obtained between high-risk and low-risk groupings according to the method of constructing the 0 or 1 matrix described above. To identify the functional clusters, the WGCNA was applied. As can be seen from **Figure 8A**, when the soft threshold equals to 4, *R* ^2^ > 0.8 and mean connectivity <<100, which means the network we construct resembles the true biological networks. Meanwhile, the tree diagram is drawn in **Supplementary Figure S4A**. **Figure 8B** presented the module–trait relationships, revealing that the blue module possesses the highest correlation (*Cor* = −0.44, *p* = 3e-16 for the high-risk group; *Cor* = 0.44, *p* = 3e-16 for the low-risk group). Moreover, the blue module exhibited a high Cox correlation in **Supplementary Figure S4B** (*Cor* = 0.86, *p* < 1e-200). Last, the 7 DECIRGs pairs and the 1,045 gene pairs contained in the blue module (**Supplementary Table S9**) are intersected to obtain the two key DECIRGs pairs (**Supplementary Figure S4C**), which are *LCK|APBA2* and *ACACA|RABEP1*.

As is evident from the IHC images (**Figure 8C**), the expression of *APBA2*, *ACACA*, and *RABEP1* in BC tissues was significantly higher than in the adjacent normal tissues, whereas *LCK* had not. RT-PCR was applied for further clinical validation for BC patients to quantify the mRNA expression levels. The results of qRT-PCR revealed that in comparison with adjacent normal breast tissues, the four key DECIRGs were all upregulated in BC tissues (**Figure 8D**, *p* < 0.05).

## 4 Discussion

The clinical application of chemotherapy has been the mainstay of the treatment of BC for a long time. Currently, BC has a 5-year survival rate close to 90%, far exceeding that of other types of tumors (18). Nonetheless, chemoresistance is an intractable problem and a clinical dilemma (3). After treatment with chemotherapy agents, BC patients with resistant tumors have limited improvement in progression-free survival, which is an unmet clinical treatment need.

On the other hand, the number of studies about immune infiltration in the tumor immune microenvironment has increased dramatically. For example, Zhang et al. explored the immune signature of BC and constructed a risk model to predict patient outcomes (19). In 2020, the infiltration of immune cells in 1109 BC samples was evaluated by Shen et al., and 11 long noncoding RNAs (lncRNAs) were identified to construct a signature (20). Unfortunately, studies simultaneously evaluating immune infiltration and chemoresistance are completely absent. Accordingly, the present study studied biomarkers relevant to chemosensitivity and immunity to build a risk model.

The strengths herein are as follows. First, immune-related genes were obtained by combining ssGSEA with unsupervised hierarchical clustering analysis instead of downloading them directly from the ImmPort database. Moreover, the TME, intratumoral immune cell content, and *HLA* genes were analyzed to investigate the heterogeneity of the groups divided by the ssGSEA score. The results showed that the grouping approach was sensible and trustworthy. Next, the cornerstones of our model are not the single DECIRGs but the paired DECIRGs obtained through cyclical pairs and iterative loops. The expression matrix, composed of only 0 or 1, focuses only on the relative expression level of the genes, without considering the sources of the data. Thus, it does not need to struggle with batch correction and detect the specific expression values of every DECIRG. Last, for the selection method of the cutoff criteria to generate the high-risk group and low-risk group, the point with the largest sum of sensitivity and specificity on the maximum AUC ROC curve was selected instead of simply choosing the median of the risk score as the standard.

After the various dimensions were evaluated in the risk model, the patients were distributed into a high-risk pathological group, such as GGI-high, chemo-insensitive, and ER-negative. The risk model was further linked to the clinical features to generate the nomogram, in which the risk score and the RCB were included as independent risk factors. Since the C-index is 0.830, the nomogram offers advantages in practicality. The concept of RCB, synthetic evaluation of the tumor bed in tumors and regional lymph nodes, was suggested by the MD Anderson Cancer Center. Its advantages in measuring the response to neoadjuvant treatment have been demonstrated for a long time through clinical practice. A retrospective study in 2007 revealed that RCB acted as a significant predictor of distant relapse-free survival, and it can be used to define categories of near-complete response and chemotherapy resistance (21). In a large BC study, RCB accurately predicted long-term survival after neoadjuvant chemotherapy in all three phenotypic subsets of BC (22). Indeed, from the nomogram, ROC curves, and the decision curve plots in **Figure 6**, it was noted that even though the percentage of the risk score was not as high as that of RCB in the nomogram, the AUC value and its decision curve distribution did not appear to differ from that of RCB. All these results highlight the value of the nomogram linking the risk score with the RCB, which is likely to bring about a new perspective for developing novel scoring systems.

Furthermore, the risk score and the immune checkpoints maintained an adequate correlation to support the efficacy of our modeling algorithms. It is generally known that immune checkpoints are negative regulators of the immune system. Among them, *PDCD1, CTLA-4, LAG3*, and *IDO1* are inhibitory checkpoint molecules, while *CD27* is a stimulatory checkpoint molecule. Their increased expression has been proven to have adverse associations with tumor outcomes (23–27). Other observations regarding the associations of the risk score with the TMB and chemotherapy agents are discussed in the *Results* section.

Although numerous strengths exist, there are also some shortcomings in the study. Because the RCB-related data were unavailable, we could not build a nomogram constructed based on the RCB and risk score in TCGA. Despite the fact that the high accuracy of the nomogram has been proven by the C-index, ROC curves, calibration curves, and DCA, the inherent flaws of the data resulted in a partial reduction of the trustworthiness. Furthermore, in the experimental validation part, we only focused on the expression level of hub DECIRGs pairs, without delving into the deep mechanism of their influence on the chemotherapy response and prognosis of BC patients. As such, further prospective and large-scale studies are warranted in the future.

Altogether, the risk score model built from seven pairs of DECIRGs has potential predictive value. More importantly, the nomogram constructed by the risk and RCB score has important practical implications.

## Data Availability Statement

Publicly available datasets were analyzed in this study. The datasets generated and/or analyzed during the current study are available in the GEO repository (https://www.ncbi.nlm.nih.gov/geo/query/acc.cgi?acc=GSE25055) and TCGA datasets (https://portal.gdc.cancer.gov/legacy-archive/search/f).

## Author Contributions

F-FD and M-QY collected and initially screened the data. WH, MH and Y-XC guided the research ideas of the full text. Z-MD performed a visual analysis of the data and was the main contributor to the manuscript. All authors contributed to the article and approved the submitted version.

## Funding

This work was supported by the Key Research and Development Program of Hubei Province under Grant number 2020BCB023, Graduate Credit Program of Wuhan University under Grant number 413000206, Education and Teaching Reform Research Project of Wuhan University under Grant number 413200095, and Young Teacher Qualification Project of the Fundamental Research Funds for the Central Universities under Grant number 2042020kf0088.

## Conflict of Interest

The authors declare that the research was conducted in the absence of any commercial or financial relationships that could be construed as a potential conflict of interest.

## Publisher’s Note

All claims expressed in this article are solely those of the authors and do not necessarily represent those of their affiliated organizations, or those of the publisher, the editors and the reviewers. Any product that may be evaluated in this article, or claim that may be made by its manufacturer, is not guaranteed or endorsed by the publisher.
