# Supplementary material for: Immune-Related Genes to Construct a Novel Prognostic Model of Breast Cancer: A Chemosensitivity-Based Study
Source: Front Immunol. 2021 Oct 26;12:734745. doi: 10.3389/fimmu.2021.734745 (PMC8576363; doi:10.3389/fimmu.2021.734745)
Supplement: Supplementary file 2 [file DataSheet_1.docx]

**Immune-Related Gene to Construct Novel Prognostic Model in Breast Cancer: A Chemosensitivity-Based Study**

Zhi-Min Deng^1#^, Wei Hu^2#^, Fang-Fang Dai^1^, Meng-Qin Yuan^1^, Min Hu^1*^, Yan-Xiang Cheng^1*^

^1^Department of Obstetrics and Gynecology, Renmin Hospital of Wuhan University, Wuhan, Hubei 430060;

^2^Department of Ultrasound, Renmin Hospital of Wuhan University, Wuhan, Hubei 430060;

^#^Contributed equally to this work

^*^***Corresponding author:***

Dr Min Hu, Department of Obstetrics and Gynecology, Renmin Hospital of Wuhan University; Wuhan, Hubei 430060; P.R. China;

E-mail: [huminmmmmm@whu.edu.cn](mailto:huminmmmmm@whu.edu.cn)

Dr Yan-Xiang Cheng, Department of Obstetrics and Gynecology, Renmin Hospital of Wuhan University; Wuhan, Hubei 430060; P.R. China;

E-mail: [rm001050@whu.edu.cn](mailto:rm001050@whu.edu.cn)

**Fig. S1.** The supplementary results of unsupervised hierarchical clustering analysis. (**A)** The tracking plots. (**B**) The delta area plot.

**Fig. S2.** **(A)** The vertical lines denote the optimal values of the penalty parameter λ. **(B)** The LASSO coefficient profiles of the DECIRGs pairs. **(C)** The heatmap of the grouping condition of GSE25055. **(D)** The survival status scatters the plot of each case of the training set. **(E)** The heatmap of the grouping condition of the validation set. **(F)** The survival status scatters plot of each case of the validation set.

**Fig. S3.** The scatter diagrams show the association between the riskscore and the **(A)** T stage, **(B)** N stage, **(C)** FIGO stage, **(D)** tumor grade, **(E)** phenotype of PR, **(F)** phenotype of ER, **(G)** mutation of ESR1, **(H)** phenotype of HER2, **(I)** Pam50 subtype, **(J)** GGI grade, **(K)** response to chemotherapy and **(L)** the immunity groupings.

**Fig. S4.** **(A)** The cluster dendrogram of the co-expression modules. **(B)** The scatter diagram of correlation between the module membership and gene significance in blue module. **(C)** The Venn diagram showing the intersection between the 7 DECIRGs and the 1045 gene pairs contained in the blue module.


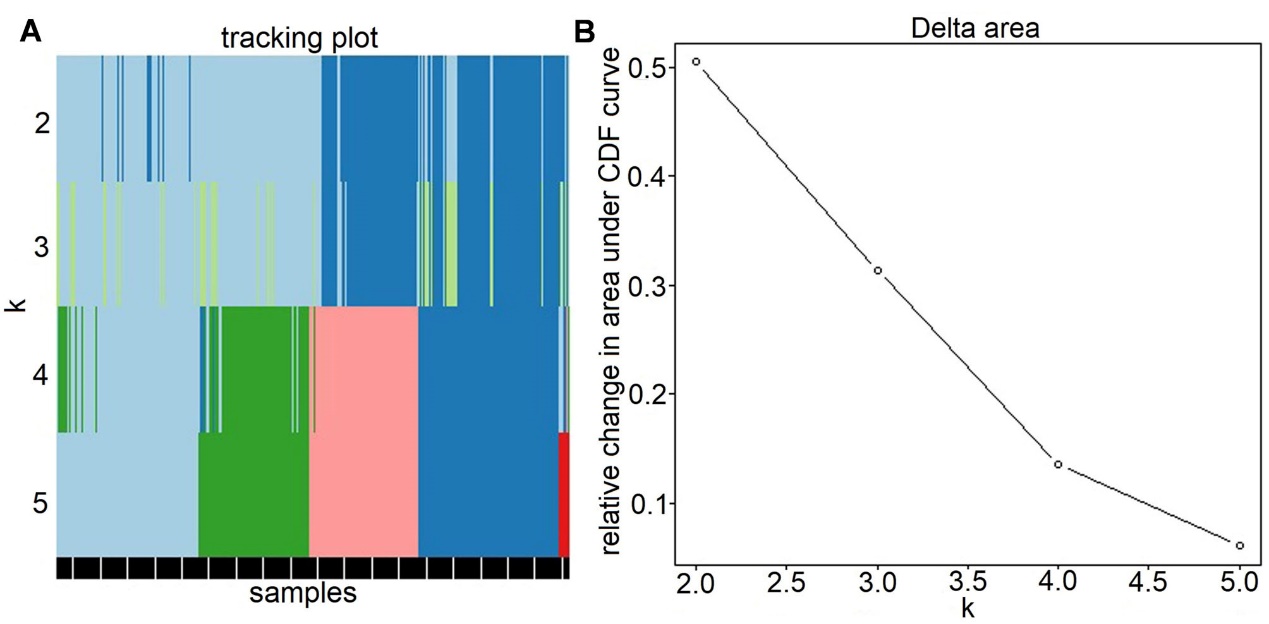


**Fig. S1**

**
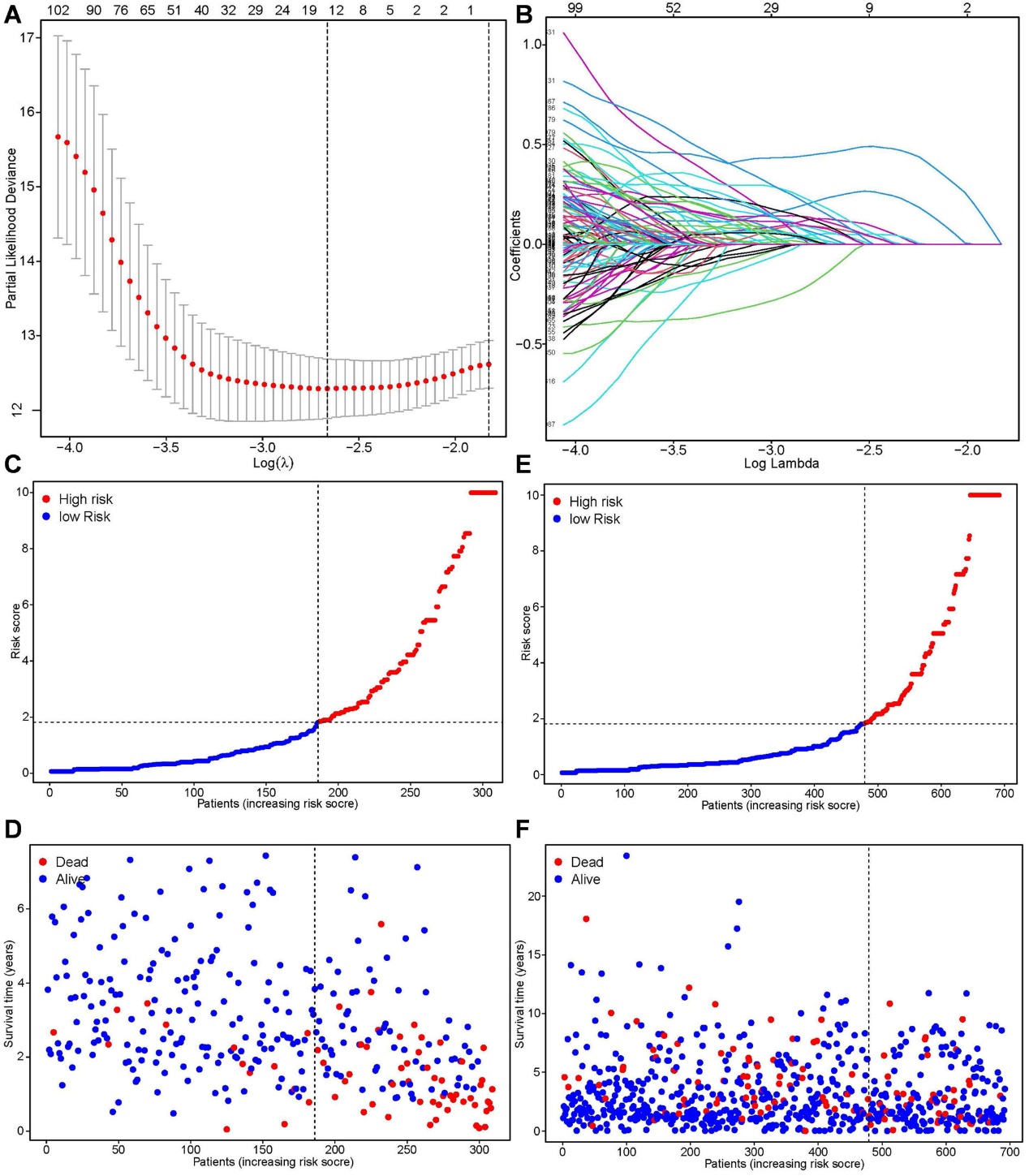
**

**Fig. S2**

**
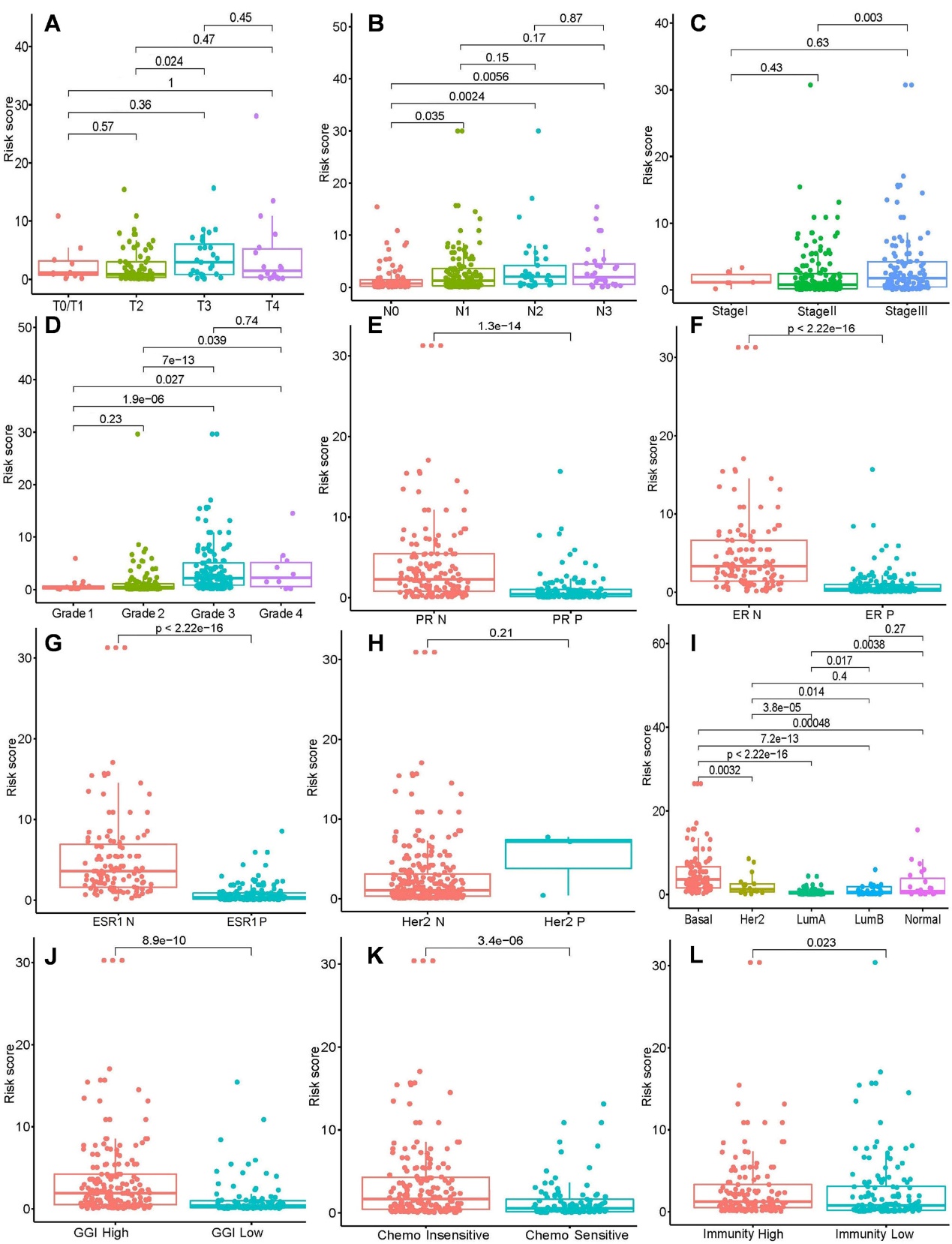
**

**Fig. S3**

**
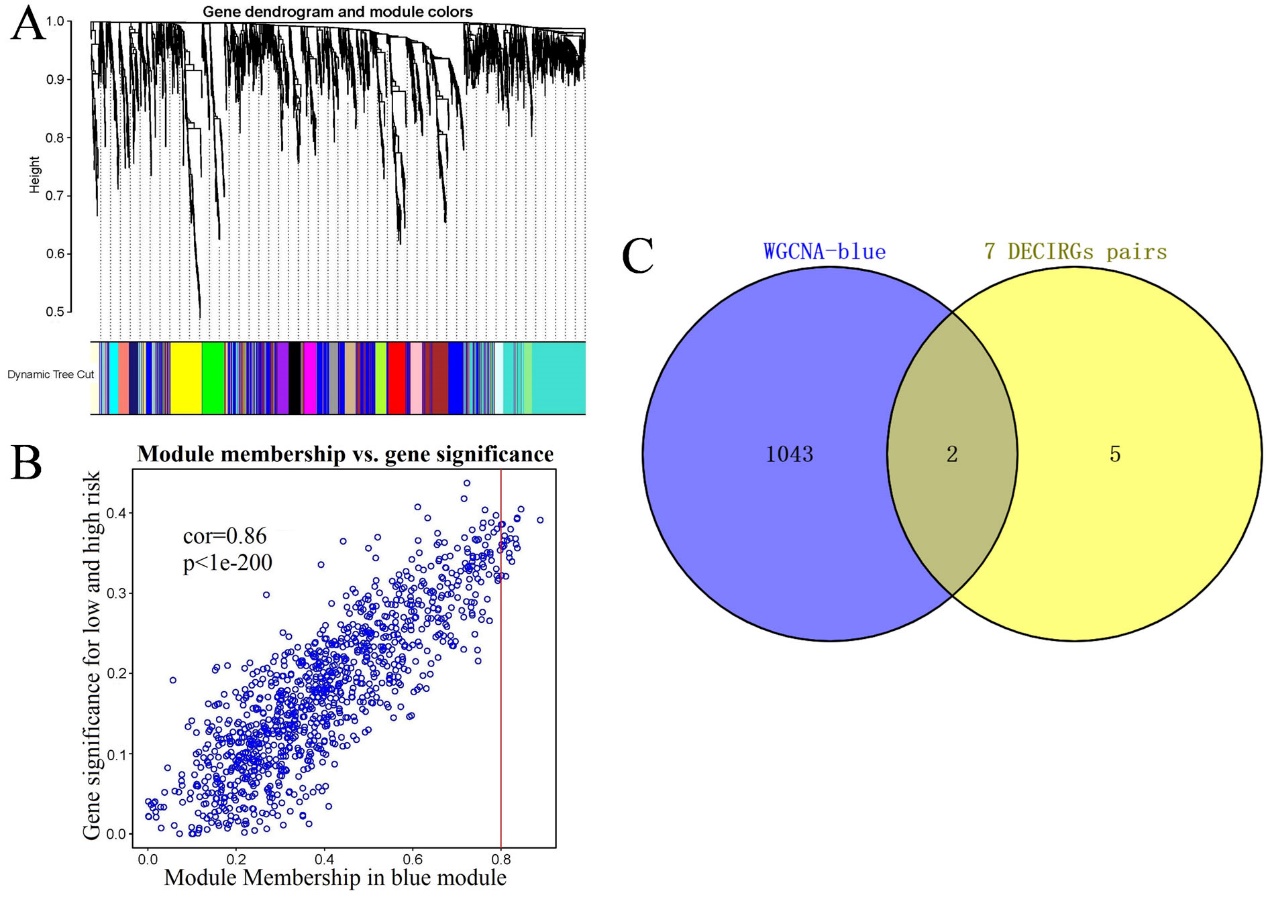
**

**Fig. S4**

**Table S1.** The primers for target gene pairs and internal reference gene.

**Table S2.** GSEA enrichment in GSE25055 based on the chemosensitivity.

**Table S3.** The immune infiltration level for 5 samples, including 29 immune cell types.

**Table S4.** The 135 DECIRGs in GSE25055.

**Table S5.** The GO analysis results of 135 DECIRGs in GSE25055 (only top 8 items).

**Table S6.** The KEGG analysis results of 135 DECIRGs in GSE25055.

**Table S7.** The DECIRG pairs preliminarily screened by LASSO regression.

**Table S8.** The detail values of univariate and multivariate Cox regression analysis.

**Table S9.** List of those gene pairs in blue modules identify by WGCNA.

**Table S1.** The primers for target gene pairs and internal reference gene.

| Gene Symbol | Forward Primer | Reverse Primer |
| --- | --- | --- |
| LCK | 5'‐GAGCTGGGACCCCCTATTTT‐3' | 5'‐CCATCCAGTCATCTTCCGGG‐3' |
| APBA2 | 5'‐GGGCCTGAAGAACCAGACACA‐3' | 5'‐TGGACTATCTTCTCGTGGGCTG‐3' |
| ACACA | 5'‐GCACAATCCTTAGGGACAACATAC‐3' | 5'‐ATGCCAATCTCATTTCCTCCTG‐3' |
| RABEP1 | 5'‐GACTATGAGCACCAGTTCCACCT‐3' | 5'‐TCTTCAGCCTCTGTCAGTTTATCC‐3' |
| GAPDH | 5'‐GGAAGCTTGTCATCAATGGAAATC‐3' | 5'‐TGATGACCCTTTTGGCTCCC‐3' |

**Table S2.** GSEA enrichment in GSE25055 based on chemosensitivity.

| **Description** | **Size** | **ES** | **NES** | **adj. *p*** | **Leading_edge** |
| --- | --- | --- | --- | --- | --- |
| Graft-versus-host disease | 36 | 0.75586 | 2.51942 | 2.98E-10 | tags=86%, list=18%, signal=71% |
| Primary immunodeficiency | 34 | 0.73573 | 2.43983 | 5.80E-09 | tags=53%, list=8%, signal=49% |
| Intestinal immune network for IgA production | 40 | 0.72859 | 2.51185 | 1.82E-10 | tags=72%, list=17%, signal=60% |
| Allograft rejection | 33 | 0.72487 | 2.39388 | 3.34E-08 | tags=79%, list=18%, signal=65% |
| Asthma | 26 | 0.70626 | 2.22182 | 6.65E-06 | tags=73%, list=20%, signal=59% |
| Autoimmune thyroid disease | 46 | 0.69372 | 2.44640 | 5.07E-09 | tags=80%, list=18%, signal=66% |
| Viral protein interaction with cytokine and cytokine receptor | 86 | 0.62212 | 2.43304 | 1.00E-10 | tags=64%, list=20%, signal=51% |
| Type I diabetes mellitus | 39 | 0.59594 | 2.03384 | 6.98E-05 | tags=67%, list=20%, signal=54% |
| Hematopoietic cell lineage | 91 | 0.58584 | 2.31136 | 1.49E-10 | tags=59%, list=20%, signal=48% |
| Inflammatory bowel disease | 59 | 0.57169 | 2.10269 | 4.69E-06 | tags=49%, list=17%, signal=41% |
| Viral myocarditis | 54 | 0.54406 | 1.97849 | 7.29E-05 | tags=50%, list=18%, signal=41% |
| Rheumatoid arthritis | 80 | 0.53557 | 2.06926 | 5.33E-07 | tags=44%, list=16%, signal=37% |
| Leishmaniasis | 69 | 0.53332 | 2.00520 | 1.21E-05 | tags=46%, list=20%, signal=37% |
| Cytokine-cytokine receptor interaction | 246 | 0.52037 | 2.40289 | 1.16E-08 | tags=55%, list=20%, signal=45% |
| Toll-like receptor signaling pathway | 93 | 0.52009 | 2.07248 | 8.02E-06 | tags=40%, list=18%, signal=33% |
| Olfactory transduction | 71 | 0.51680 | 1.95143 | 0.000115 | tags=44%, list=20%, signal=35% |
| Antigen processing and presentation | 65 | 0.51321 | 1.91985 | 0.000694 | tags=45%, list=18%, signal=37% |
| Taste transduction | 61 | 0.50722 | 1.88906 | 0.002133 | tags=46%, list=22%, signal=36% |
| NF-kappa B signaling pathway | 93 | 0.50571 | 2.01521 | 1.84E-05 | tags=55%, list=26%, signal=41% |
| Natural killer cell mediated cytotoxicity | 111 | 0.50125 | 2.06644 | 7.29E-06 | tags=44%, list=18%, signal=36% |
| Cytosolic DNA-sensing pathway | 51 | 0.49862 | 1.79779 | 0.007513 | tags=39%, list=17%, signal=33% |
| Malaria | 44 | 0.49741 | 1.73296 | 0.015564 | tags=55%, list=22%, signal=43% |
| Staphylococcus aureus infection | 71 | 0.48802 | 1.84277 | 0.000758 | tags=54%, list=20%, signal=43% |
| Cell adhesion molecules | 122 | 0.45981 | 1.92742 | 5.84E-05 | tags=52%, list=22%, signal=40% |
| Influenza A | 156 | 0.45097 | 1.95694 | 2.16E-06 | tags=31%, list=17%, signal=26% |
| Th1 and Th2 cell differentiation | 83 | 0.44822 | 1.74043 | 0.001427 | tags=27%, list=12%, signal=24% |
| Chemokine signaling pathway | 167 | 0.44665 | 1.96770 | 4.22E-06 | tags=32%, list=14%, signal=28% |
| Pertussis | 65 | 0.43968 | 1.64481 | 0.020248 | tags=42%, list=21%, signal=33% |
| Tuberculosis | 161 | 0.43310 | 1.89207 | 1.42E-05 | tags=35%, list=20%, signal=29% |
| T cell receptor signaling pathway | 98 | 0.43179 | 1.73378 | 0.001404 | tags=28%, list=13%, signal=24% |
| Measles | 128 | 0.42058 | 1.78390 | 0.000756 | tags=38%, list=22%, signal=30% |
| B cell receptor signaling pathway | 76 | 0.41686 | 1.59522 | 0.012731 | tags=37%, list=19%, signal=30% |
| Th17 cell differentiation | 97 | 0.41172 | 1.66001 | 0.006409 | tags=37%, list=21%, signal=30% |
| Neuroactive ligand-receptor interaction | 295 | 0.39369 | 1.85805 | 2.25E-07 | tags=35%, list=17%, signal=29% |
| TNF signaling pathway | 101 | 0.39149 | 1.58451 | 0.015564 | tags=32%, list=17%, signal=27% |
| Epstein-Barr virus infection | 191 | 0.39146 | 1.75161 | 6.81E-05 | tags=31%, list=18%, signal=26% |
| Phagosome | 132 | 0.38964 | 1.65378 | 0.001955 | tags=36%, list=23%, signal=28% |
| Coronavirus disease - COVID-19 | 201 | 0.387046 | 1.73793 | 9.98E-05 | tags=30%, list=23%, signal=23% |
| Osteoclast differentiation | 121 | 0.37210 | 1.55282 | 0.020248 | tags=27%, list=15%, signal=23% |
| NOD-like receptor signaling pathway | 145 | 0.35091 | 1.50858 | 0.020312 | tags=32%, list=20%, signal=26% |
| Neutrophil extracellular trap formation | 145 | 0.34572 | 1.48627 | 0.027793 | tags=23%, list=14%, signal=20% |
| Kaposi sarcoma-associated herpesvirus infection | 176 | 0.32383 | 1.43777 | 0.028573 | tags=24%, list=19%, signal=20% |
| Estrogen signaling pathway | 117 | -0.40167 | -1.59108 | 0.020248 | tags=23%, list=11%, signal=21% |
| Peroxisome | 71 | -0.43623 | -1.58404 | 0.020248 | tags=31%, list=17%, signal=26% |
| Drug metabolism - other enzymes | 55 | -0.47862 | -1.66586 | 0.028573 | tags=25%, list=8%, signal=23% |
| Metabolism of xenobiotics by cytochrome P450 | 51 | -0.49282 | -1.69545 | 0.030652 | tags=29%, list=9%, signal=27% |
| Drug metabolism - cytochrome P450 | 49 | -0.49695 | -1.70286 | 0.021633 | tags=24%, list=9%, signal=22% |
| Fatty acid elongation | 17 | -0.72533 | -1.96849 | 0.006409 | tags=35%, list=13%, signal=31% |

**Note**: ES, enrichment score; NES, Normalized Enrichment Score; adj. *p*, adjustment *p*-value. The results in the “ES” and “NES” columns with five decimal places.

**Table S3.** The immune infiltration level for 5 samples, including 29 immune cell types.

| **Cell type** | **GSM615096** | **GSM615097** | **GSM615103** | **GSM615114** | **GSM615120** |
| --- | --- | --- | --- | --- | --- |
| aDCs | 1.36304 | 1.35626 | 1.24905 | 1.33185 | 1.33560 |
| APC_co_inhibition | 1.09734 | 1.36563 | 1.20402 | 1.25872 | 1.15599 |
| APC_co_stimulation | 1.30681 | 1.33045 | 1.29140 | 1.33542 | 1.31889 |
| B_cells | 1.14643 | 1.23333 | 1.21523 | 1.18364 | 1.23729 |
| CCR | 1.32177 | 1.36221 | 1.33203 | 1.35657 | 1.35457 |
| CD8+_T_cells | 1.56441 | 1.44981 | 1.31090 | 1.44372 | 1.48265 |
| Check-point | 1.28179 | 1.33709 | 1.29963 | 1.32862 | 1.30966 |
| Cytolytic_activity | 1.33008 | 1.23178 | 1.26318 | 1.24232 | 1.49677 |
| DCs | 1.24768 | 1.21412 | 1.18724 | 1.29347 | 1.24910 |
| HLA | 1.64194 | 1.75938 | 1.69037 | 1.68734 | 1.74821 |
| iDCs | 1.25921 | 1.50093 | 1.38390 | 1.41081 | 1.35717 |
| Inflammation-promoting | 1.42265 | 1.43756 | 1.36466 | 1.40421 | 1.48932 |
| Macrophages | 1.61141 | 1.63781 | 1.59566 | 1.65152 | 1.61987 |
| Mast_cells | 1.33118 | 1.46453 | 1.40754 | 1.19558 | 1.26090 |
| MHC_class_I | 1.84779 | 1.92067 | 1.88331 | 1.91262 | 1.95391 |
| Neutrophils | 1.35797 | 1.41477 | 1.33875 | 1.40236 | 1.41491 |
| NK_cells | 1.20510 | 1.21877 | 1.01499 | 1.09694 | 1.19412 |
| Parainflammation | 1.53782 | 1.59172 | 1.60887 | 1.59878 | 1.63043 |
| pDCs | 1.34915 | 1.39664 | 1.35408 | 1.37255 | 1.40789 |
| T_cell_co-inhibition | 1.18792 | 1.19814 | 1.18100 | 1.23315 | 1.16887 |
| T_cell_co-stimulation | 1.22681 | 1.26956 | 1.24325 | 1.23877 | 1.25681 |
| T_helper_cells | 1.42256 | 1.33827 | 1.25122 | 1.22756 | 1.53595 |
| Tfh | 1.31378 | 1.30061 | 1.18238 | 1.35633 | 1.25917 |
| Th1_cells | 1.28821 | 1.30567 | 1.31796 | 1.33852 | 1.26288 |
| Th2_cells | 1.39949 | 1.45818 | 1.44180 | 1.46756 | 1.41604 |
| TIL | 1.31973 | 1.37231 | 1.31273 | 1.31413 | 1.35394 |
| Treg | 1.47725 | 1.49514 | 1.45994 | 1.47502 | 1.48575 |
| Type_I_IFN_Reponse | 1.45910 | 1.56454 | 1.55501 | 1.49886 | 1.57748 |
| Type_II_IFN_Reponse | 1.64926 | 1.82187 | 1.50020 | 1.75052 | 1.71553 |

**Note**: All the results with five decimal places.

**Table S4.** The 135 DECIRGs in GSE25055.

| **Genes** | | | | | | | |
| --- | --- | --- | --- | --- | --- | --- | --- |
| AREG | MNAT1 | IGFBP4 | STK17B | CD3D | CFAP69 | CCR3 | SCCPDH |
| TJP3 | MAGED2 | CD53 | ACOX2 | CDC42BPB | LRMP | SYT1 | ESR1 |
| PRRC2B | RABEP1 | ATP13A2 | PBX1 | IKZF1 | RUBCNL | THBS1 | PGR |
| CTDSPL | AGR2 | LAMA3 | LCK | ATR | NPDC1 | ITGA4 | SLC2A10 |
| PIK3CD | GSTZ1 | UGDH | DNALI1 | MFNG | TMCO3 | MED1 | GRP |
| ERBB3 | APBA2 | ALCAM | KRT18 | CA12 | ARHGAP25 | EPN3 | SCNN1A |
| SLC39A6 | CUEDC1 | CD151 | NAT1 | P2RY14 | CORO1A | IL32 | SYT17 |
| ATP9A | LMAN2L | MYB | DNPEP | DST | GOLGA2 | ARRB2 | IGF2BP2 |
| DLG5 | NFE2L1 | DNAJC12 | GPR171 | PIGN | THEMIS2 | PIP | POU2AF1 |
| TFF1 | MAPT | TBC1D9 | EVI2B | TANC2 | PTPRC | IFT74 | HCAR3 |
| MSN | LTBP3 | EFHC1 | GPR18 | FLNB | GIMAP4 | COBL | NTM |
| VAV3 | PEX11A | TCEA2 | SERPINA3 | GPR183 | GFRA1 | LILRB2 | GMFG |
| RASSF2 | CSF2RB | ADCY9 | ITGA3 | CYTIP | CCL5 | AZGP1 | VGLL1 |
| IRX5 | BARD1 | CD69 | LOC100130449 | GZMB | SELL | PSD3 | SRGN |
| PRLR | PPP1R26 | CD27 | STAP1 | ST8SIA4 | REEP1 | ANO1 | TRAT1 |
| TTC39A | PSMB9 | LRIG1 | ACACA | WASL | SCUBE2 | GSTT2 | IGHM |
| GREB1 | PCYOX1 | S100A4 | KLRB1 | CCND1 | NVL | GZMM |  |

**Table S5.** The GO analysis results of 135 DECIRGs in GSE25055.

| GO term | Gene symbol | *adj. p* |
| --- | --- | --- |
| T cell activation | PIK3CD/DLG5/MSN/PRLR/CD151/MYB/  CD27/LCK/GPR18/CD3D/GPR183/  CORO1A/PTPRC/CCL5/LILRB2 | 0.001603 |
| leukocyte migration | PIK3CD/MSN/VAV3/LCK/GPR18/ITGA3/  STAP1/GPR183/WASL/CORO1A/CCL5/  SELL/THBS1/ITGA4/IGHM | 0.001986 |
| lymphocyte differentiation | PIK3CD/MYB/CD27/LCK/GPR18/  CD3D/IKZF1/MFNG/GPR183/  PTPRC/ITGA4/LILRB2 | 0.004559 |
| positive regulation of leukocyte activation | VAV3/MYB/CD27/LCK/STAP1/  GPR183/CORO1A/PTPRC/CCL5/  THBS1/LILRB2/IGHM | 0.006445 |
| positive regulation of cell activation | VAV3/MYB/CD27/LCK/STAP1/  GPR183/CORO1A/PTPRC/CCL5/  THBS1/LILRB2/IGHM | 0.006445 |
| leukocyte cell-cell adhesion | DLG5/MSN/MYB/CD27/LCK/CORO1A/  PTPRC/CCL5/SELL/ITGA4/LILRB2 | 0.006445 |
| positive regulation of antigen receptor-mediated signaling pathway | LCK/STAP1/PTPRC/TRAT1 | 0.006445 |
| positive regulation of cell adhesion | VAV3/AGR2/MYB/CD27/LCK/  ITGA3/CORO1A/PTPRC/CCL5/  ITGA4/IFT74/LILRB2 | 0.006445 |

**Notes**: Only the top 8 were presented in the table**.**

**Table S6.** The KEGG analysis results of 135 DECIRGs in GSE25055.

| KEGG term | Gene symbol | *adj. p* |
| --- | --- | --- |
| Focal adhesion | PIK3CD/VAV3/LAMA3/ITGA3/  FLNB/CCND1/THBS1/ITGA4 | 0.025483647 |
| Proteoglycans in cancer | PIK3CD/ERBB3/MSN/VAV3/  FLNB/CCND1/THBS1/ESR1 | 0.025483647 |
| PI3K-Akt signaling pathway | AREG/PIK3CD/ERBB3/PRLR/  LAMA3/MYB/ITGA3/CCND1  /THBS1/ITGA4 | 0.037637049 |
| Estrogen signaling pathway | PIK3CD/TFF1/ADCY9/  KRT18/ESR1/PGR | 0.045928933 |
| Endocrine resistance | PIK3CD/ADCY9/CCND1/  MED1/ESR1 | 0.049801441 |

**Table S7.** The DECIRG pairs preliminarily screened by LASSO regression.

| DECIRG pairs | | |
| --- | --- | --- |
| LCK\|APBA2 | CCL5\|PRLR | PSMB9\|NAT1 |
| CD69\|DNAJC12 | MSN\|CD151 | MSN\|NFE2L1 |
| PIGN\|ADCY9 | PIGN\|GREB1 | ITGA4\|LAMA3 |
| ITGA4\|DNAJC12 | ITGA4\|NAT1 | IGF2BP2\|IFT74 |
| ST8SIA4\|PSD3 | GOLGA2\|ALCAM | PEX11A\|GREB1 |
| CTDSPL\|MAPT | ACACA\|RABEP1 | ACOX2\|PIP |
| ACOX2\|AREG | ACOX2\|DNALI1 |  |

**Table S8.** The detailed values of univariate and multivariate Cox regression analysis.

| id | Univariate analysis | | | | Multivariate analysis | | | |
| --- | --- | --- | --- | --- | --- | --- | --- | --- |
|  | **HR** | **HR.95L** | **HR.95H** | ***p*** | **HR** | **HR.95L** | **HR.95H** | ***p*** |
| Age | 1.01 | 0.99 | 1.04 | 0.31 | 1.00 | 0.98 | 1.03 | 0.78 |
| T | 1.45 | 1.08 | 1.93 | 0.01 | 1.01 | 0.70 | 1.45 | 0.97 |
| N | 1.76 | 1.38 | 2.25 | 6.50E-06 | 1.43 | 0.95 | 2.16 | 0.08 |
| Grade | 1.78 | 1.19 | 2.67 | 4.69 E-03 | 1.41 | 0.78 | 2.53 | 0.25 |
| Stage | 2.37 | 1.43 | 3.93 | 7.76E-04 | 0.93 | 0.39 | 2.22 | 0.86 |
| ER | 0.35 | 0.21 | 0.60 | 1.10E-04 | 1.21 | 0.35 | 4.23 | 0.76 |
| PR | 0.44 | 0.25 | 0.77 | 3.77E-03 | 1.07 | 0.48 | 2.39 | 0.87 |
| HER2 | 2.08 | 0.29 | 15.04 | 0.47 | 0.53 | 0.06 | 4.59 | 0.56 |
| ESR1 | 0.34 | 0.20 | 0.58 | 6.99E-05 | 0.88 | 0.27 | 2.89 | 0.84 |
| GGI | 2.87 | 1.45 | 5.67 | 2.41E-03 | 1.72 | 0.66 | 4.49 | 0.26 |
| Pam50 | 1.53 | 1.25 | 1.86 | 2.57E-05 | 1.21 | 0.80 | 1.81 | 0.37 |
| RCB | 3.43 | 2.28 | 5.16 | 2.98E-09 | 3.00 | 1.87 | 4.81 | 4.64E-06 |
| Immunity | 1.01 | 0.61 | 1.69 | 0.96 | 1.06 | 0.62 | 1.82 | 0.83 |
| Chemosensitivity | 0.21 | 0.10 | 0.44 | 3.71E-05 | 0.63 | 0.27 | 1.51 | 0.30 |
| Riskscore | 1.15 | 1.12 | 1.19 | 6.74E-21 | 1.10 | 1.06 | 1.15 | 2.83E-07 |

**Note**: All the results with 2 decimal places.

**Table S9.** List of those gene pairs in blue modules identify by WGCNA.

As shown in separate Table S9.xls.
